# A safety evaluation of allogeneic freeze-dried platelet-rich plasma or conditioned serum compared to autologous frozen products equivalents in equine healthy joints

**DOI:** 10.1186/s12917-022-03225-4

**Published:** 2022-04-18

**Authors:** Livia Camargo Garbin, Erin K. Contino, Christine S. Olver, David D. Frisbie

**Affiliations:** 1 Equine Orthopaedic Research Center, Colorado State University, 300 West Drake Road, , Fort Collins, CO 80523 USA; 2 Present Address: Department of Large Animal Medicine, College of Veterinary Medicine, University of Georgia, 501 D.W. Brooks Drive, 30602 Athens, GA USA; 3 C. Wayne McIlwraith Translational Medicine Institute, Colorado State University, 2350 Drive, Fort Collins, CO 80523 USA; 4 Veterinary Diagnostic Laboratory, Clinical Pathology Section, Department of Microbiology, Immunology, and Pathology, College of Veterinary Medicine and Biomedical Sciences, Colorado State University, Fort Collins, CO 80523 USA

**Keywords:** Conditioned serum, Platelet-rich plasma, Osteoarthritis, Allogeneic, Horse

## Abstract

**Background:**

Hemoderivatives such as autologous conditioned serum (ACS) and platelet-rich plasma (PRP) have been used as potential disease-modifying therapies in musculoskeletal disorders such as osteoarthritis (OA). These therapies are based on the delivery of multiple growth factors and anti-inflammatory cytokines that are known to participate in inflammatory processes. The variability of cytokine content due to the autologous nature of the product, the non-availability for immediate use and need for storage at low temperatures are limitations for its use in the field. An allogeneic freeze-dried conditioned serum (CS) and PRP would provide field clinicians with a more practical approach to use such products in daily practice. Based on in vitro preliminary data, this experimental study aimed to test the in vivo safety of allogeneic freeze-dried CS and PRP in healthy joints, using the horse as a model.

**Results:**

Eight horses were randomly assigned and treated with PRP or CS. Horses had three joints injected with ALLO-FD PRP or CS, and three contralateral joints injected with the AUTO version of the same product, by a blinded clinician. Horses were evaluated clinically, and had synovial fluid collected at different time points and evaluated for cell content, PGE_2_ and protein. Both CS and PRP products triggered a self-limiting and mild inflammatory response in equine healthy joints. This was indicated by the transient increase in nucleated cell count, PGE_2_ and total protein in synovial fluid. This mild inflammatory response did not result in significant lameness and was not different among the groups.

**Conclusions:**

The allogeneic freeze-dried PRP and CS showed to be overall safe and not dissimilar compared to their autologous frozen version in equine healthy joints. Further studies are necessary to evaluate the modulatory effects of these therapies in a clinical setting.

**Supplementary Information:**

The online version contains supplementary material available at 10.1186/s12917-022-03225-4.

## Background

Potentially disease-modifying therapies, such as platelet-rich plasma (PRP) and autologous conditioned serum (ACS), have been used experimentally and clinically as an option for the treatment of musculoskeletal disorders such as osteoarthritis (OA) [1, 2]. Due to the delivery of multiple growth factors [3] and anti-inflammatory cytokines [4, 5] both PRP [6–8] and ACS [2, 9] demonstrated promising outcomes in early OA, resulting in significant clinical improvement and improvement of osteoarthritic changes in joints [3, 5].

While positive results were seen with the clinical use of commercial ACS and PRP products, multiple hurdles preclude its widespread acceptance and use. The time required for its preparation, the need for storage at low temperatures and the significant variations in cytokine profile [10] due to its autologous nature are critical barriers to widespread acceptance.

The creation of an allogeneic freeze-dried conditioned serum (CS) and PRP would be beneficial in reducing limitations for use of these biologics in daily practice. Such products would offer a more stable, homogeneous and quantified product option for use. Our research group has demonstrated through in vitro proof-of-principle studies that lyophilization of biologic products such as PRP [11] preserves protein integrity and biologic activity [12, 13]. Further, that allogeneic biologic products demonstrated equivalent results compared to autologous preparations [11]. The purpose of the study was to test the safety of an allogeneic freeze-dried version of PRP and CS compared to the current preparations used in the field (frozen autologous), using the horse as a model.

Based on in vitro experiments, we hypothesized that allogeneic freeze-dried PRP and CS would produce similar effects on the joints compared to autologous frozen preparations.

## Results

### Blood collection and platelet-rich plasma creation

Blood used in this study and platelet-derived product were submitted for complete blood count (CBC) analysis. Platelet count observed was 112.5 × 10^3^ platelets/µL for whole blood but platelet clumps were common. Values for red blood cells (RBC), nucleated cell count (NCC) and differential NCC were all within normal range (Table 1). The average count for platelet-derived products was 180.25 × 10^3^ platelets/μL, and the average number of RBC and NCC were below normal range^1^ (Table 1). Considering the 3-fold concentration of the PRP used, the estimated number of platelets was above average while NCC values were still below normal range^1^ (Table 1).

**Table 1:** Average for cell counts for whole blood and the platelet-derived products

	**Platelets × 10**^**3**^** cells/ µL**	**RBC × 10**^**3**^** cells/ µL**	**NCC × 10**^**3**^** cells/ µL**	**Lymphocytes × 10**^**3**^** cells/ µL**	**Monocytes × 10**^**3**^** cells/ µL**	**Neutrophils × 10**^**3**^** cells/ µL**
Reference Range (blood)	125–300	6.5–10.5	5.5–10.5	3–7	0–0.6	3–7
Whole blood	112.5	9.2	5.92	2.63	0.125	2.85
Platelet- product	180.25	0.0362	0.4	3.75	0	0
Platelet-rich plasma (estimated measures for 3-fold)	540	0.1087	1.2	1.125	0	0

Estimated values for platelet-rich plasma used in this study (3-fold concentration) are also represented. RBC; red blood cell, NCC: nucleated cell count. Reference values are based on normalization studies performed at the Colorado State University’s Clinical Pathology Laboratory^1^.

### Clinical examination

#### Vital parameters

There was no significant difference in heart rate or temperature over time or between treatments. Respiratory rate significantly increased (*P* = 0.006) only at T6 (24 ± 3.

breaths/min) compared to baseline (18 ± 1 breath/min), but was not different between treatments.

#### Synovial fluid gross findings

Treatments did not present any significant difference and only time had a significant effect for joint clarity (P ≤ 0.0001) and color (P ≤ 0.0001). Joint fluid was classified as hazy at T6, T24 and T48 hours, cloudy at T96 hours and clear at T168. Synovial fluid color was orange at T24 and T48, while at T96, samples were predominantly straw color, not differing significantly from T0, T6 or T168.

#### Joint parameters

Time had a significant effect (*P* = 0.031) on joint (range of motion) ROM. Following treatment, all joints had a decrease in ROM at T24 compared to T0 (*P* = 0.0078) and T6 (*P* = 0.0065). ROM increased but did not return to baseline values by T168. Joints injected with different treatments did not present significant difference in ROM (*P* = 0.9562) at any time point (*P* = 0.5272). The amount of joint effusion was only significantly influenced by time (*P* < 0.0001), with an increase in effusion at T6 (*P* < 0.0001). Joint effusion decreased significantly between T6 and T24 (*P* = 0.0089), remaining low at all time points (*P* < 0.0089), but not returning to baseline values. Effusion varied significantly depending on the joint injected (*P* = 0.0002) and the interaction of the joint and treatment injected was also significant (*P* = 0.0228). The tarsocrural and the middle carpal joints had significantly more effusion compared to the metacarpophalangeal joint (*P* < 0.0001 and *P* = 0.0018, respectively). No significance across time or treatment was found for circumference (*P* ≥ 0.3793) or heat (*P* ≥ 0.0907).

#### Lameness and flexion scores

Mean values for lameness were not different across time (*P* = 0.9038), by joint (*P* = 0.1406) or by treatment (*P* = 0.2799). The mean lameness scores values across the experiment did not vary greatly, ranging from grade 0.5 to 0.7 out of 5. Flexion scores were significantly affected by time (*P* = 0.0215), by joint (*P* < 0.001), but not by treatment (*P* = 0.1552). Horses demonstrated less discomfort on carpi flexion (score 0.46 out of 4) than metacarpophalangeal (0.8 out of 4; *P* = 0.0016) and tarsocrural joints (2 out of 4; *P* < 0.001). Across time, compared to baseline, flexion scores increased at T6 (*P* = 0.0012), T24 (*P* = 0.0122), and T168 (*P* = 0.0147).

### Synovial fluid analysis

Statistical model assumptions were not met for total protein, NCC, small mononuclear cells (SMN), or PGE_2_. For these outcomes, data was log transformed and analysis was performed again to meet the model assumptions.

#### Total protein (g/dL)

**Fig. 1:**
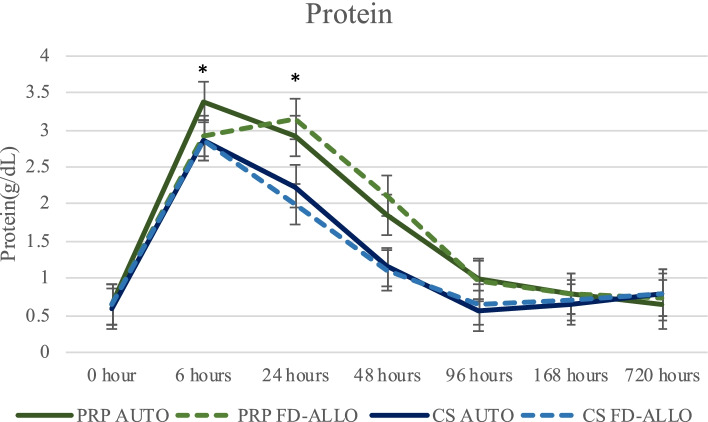
Protein levels in synovial fluid across time. Protein was measured in synovial fluid prior to (T0) and at T6, T24, T48, T96 and T168 hours after one intra-articular injection of different preparations [AUTO or FD-ALLO] of either PRP or CS. Protein levels for all groups were statistically elevated at T6 and T24 compared to other time points. Raw data is presented as mean and SEM. Log-transformed data was used for the statistical analysis with a level of significance set as *P* ≤0.05. Specific differences between groups can be observed on Supplementary Table 1. Asterisks represent the statistical difference of protein over time compared to other time points

Time, joint and their interaction had a significant effect on total protein levels (*P* ≤ 0.0001), but no effect of treatment was observed (*P* = 0.33). Carpi (2.6919 ± 0.172) had significantly higher protein compared to tarsocrural (1.7159 ± 0.1721; *P* < 0.0001) and metacarpophalangeal joints (1.4385 ± 0.172; *P* < 0.0001). There was also a difference across time (*P* ≤ 0.0001), with a mean increase in protein levels at T6 (2.9979 ± 0.1802) and T24 (3.0864 ± 0.1807), compared to other time points (*P* ≤ 0.001; Supplementary Table 1), decreasing after that but not returning to baseline values (Fig. 1).

#### Nucleated cell count [NCC (cells/ µL)]

Significant effects were found for time (*P* < 0.001), joint (*P* < 0.001), treatment group (*P* = 0.0009) and treatment joint interaction (*P* = 0.0084).

**Fig. 2:**
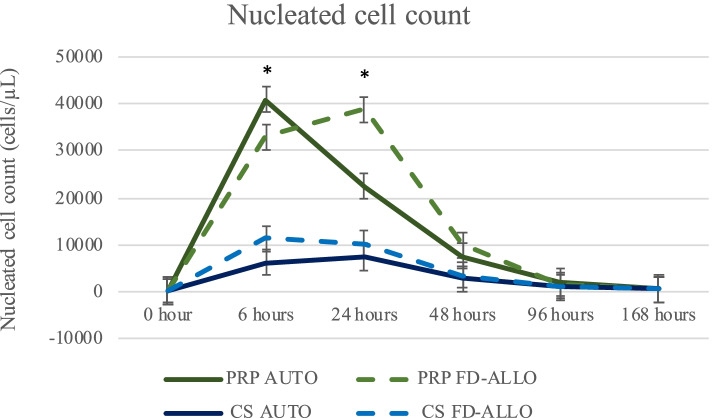
Nucleated cell count (NCC) in synovial fluid across time prior to (T0) and at T6, T24, T48, T96 and T168 hours after one intra-articular injection of autologous frozen or allogeneic freeze-dried [AUTO or FD-ALLO, respectively] of platelet-rich plasma (PRP) or conditioned serum (CS). The graph is based on raw data used for illustration purposes. Log-transformed data was used for the statistical analysis with a level of significance of *P* ≤ 0.05. Values are represented as means and bars represent standard error of the mean. Asterisks represent the statistical difference of NCC over time compared to T0

Treatment with either AUTO (10,563 ± 1579.83, *P* = 0.0116) or FD-ALLO PRP (12,103 ± 1576.07, *P* = 0.0051) resulted in higher NCC compared to AUTO CS (2597.86 ± 1579.83), but not FD- ALLO CS (3797.89 ± 1576.07, Fig. 2). Joints treated with FD-ALLO CS also had higher NCC compared to those treated with AUTO CS (*P* = 0.0005).

Across treatments, a significant increase in NCC was observed over time from T0 (202,08 ± 1492.61) through T168 (595.83 ± 1492.61) post injection (*P* ≤ 0.001), achieving the highest values at T6 (22,852 ± 1492.61) and T24 (19,765 ± 1506.49) (Fig. 2). Nucleated cells count were significantly higher in the metacarpophalangeal (7444.31 ± 1220.21; *P* ≤ 0.001) and carpi (10,443 ± 1217.43; *P* = 0.0017) compared to the tarsocrural joints (3909.04 ± 1220.21). There was a significant effect of treatment group over time (*P* ≤ 0.001; Supplementary Table 2; Fig. 2) and of treatment by joint (*P* = 0.0084).

#### Large mononuclear cells [LMN (% of NCC)]

LMN were significantly increased only by time (*P* < 0.0001) and treatment group (*P* < 0.0016). Across time, compared to T0 (47.37 ± 5.10), LMN were significantly decreased at T6 (16.68 ± 5.10; *P* < 0.001) and T24 (33.36 ± 5.28; *P* = 0.0003) and significantly increased at T96 (74.81 ± 5.10; *P* < 0.001) and T168 (76.38 ± 5.10; *P* < 0.001; Supplementary Table 3). Comparing treatment groups, LMN were significantly lower for both PRP treatments [FD-ALLO (36.97 ± 7.79) and AUTO (44.01 ± 4.79)] compared to both CS treatments [FD-ALLO (57.48 ± 4.85) and AUTO (56.45 ± 4.79); *P* ≤ 0.0276; Supplementary Table 4].

#### Polymorphonuclear cells [PMN (% of NCC)]

PMN changed over time (*P* < 0.001), treatment group (*P* < 0.0001), and by the interaction of time and treatment group (*P* < 0.0001). Regardless of treatment, PMN was significantly increased at T6 (80.50 ± 3.46), T24 (60.59 ± 3.59) and T48 (29.75 ± 3.44; *P* < 0.001) compared to baseline (4.69 ± 3.46; Supplementary Table 3). Carpi had significantly more PMN cells (32.78 ± 1.8711) compared to metacarpophalangeal joints (27.69 ± 1.8768; *P* = 0.0156). Joints treated with AUTO CS (20.42 ± 3.2) had significantly lower PMN compared to joints treated with FD-ALLO CS (24.25 ± 3.16; *P* = 0.0009) and both PRP treatments [AUTO (32.11 ± 3.6) and FD-ALLO (33.86 ± 3.16); *P* < 0.0001; Supplementary Table 4].

#### Small mononuclear cells [SMN (% of NCC)]

There was a significant effect of time (*P* < 0.001) but not of treatment group (*P* = 0.2466; Supplementary Table 4) or joint (*P* = 0.5571) on SMN. After treatment at T0, there was a significant decrease of SMN at T6 (5.31 ± 3.81; *P* = 0.0036), and levels remained significantly decreased at all time points compared to baseline (47.88 ± 3.81; *P* < 0.001; Supplementary Table 3).

#### Prostaglandin E_2_ (PGE_2_ – pg/mL)

**Fig. 3:**
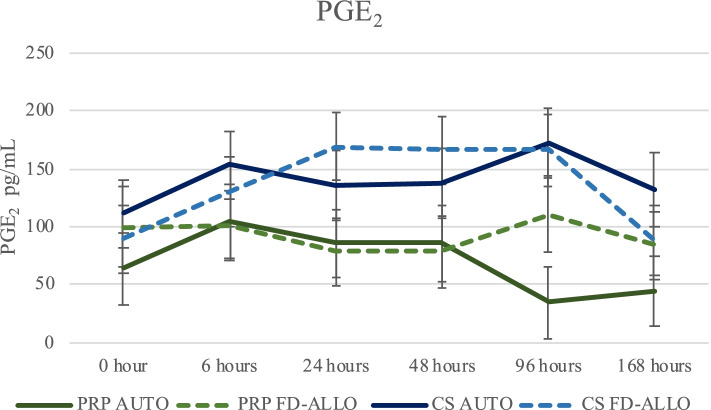
PGE_2_ levels in synovial fluid across time. PGE_2_ levels were measured in synovial fluid from joints treated with different preparations [AUTO or FD-ALLO] of either PRP or CS at different times points (T0) and at T6, T24, T48, T96 and T168 hours after the injection. Specific differences between groups can be observed on Supplementary Table 5. Raw data is presented as mean and SEM. Log-transformed data was used for the statistical analysis with a level of significance set as *P* ≤0.05

PGE_2_ did not demonstrate a statistical difference on the main effect of time or treatment group (*P* = 0.0769 and *P* = 0.1155, respectively). However, on individual comparisons, PGE_2_ increased at T6 (122.54 ± 18.28; *P* = 0.0031) and T24 hours (117.4 ± 18.01; *P* = 0.0260) post-treatment, being statistically higher compared to T168 hours (87.08 ± 18.21), but not T0 (*P* = 0.1924; Supplementary Table 5; Fig. 3). The metacarpophalangeal joint (118.93 ± 16.45) had significantly higher levels of PGE_2_ compared to the tarsocrural joint (93.72 ± 16.43; *P* = 0.0468) but not compared to carpi (114.77 ± 16.39; *P* = 0.5521).

## Discussion

The main goal of this study was to perform a safety evaluation of an allogeneic freeze-dried version of two biological therapies (PRP and CS) in equine normal joints. The most important findings in this study were that both ALLO-FD PRP and CS effects were not significantly different compared to the frozen autologous treatments. Similar to previous studies [14–16] all treatments triggered a transitory self-limiting inflammatory response within the joints, which was more pronounced on the PRP group.

This mild but noteworthy inflammatory response was evidenced by the increase in NCC, PGE_2_ and total protein. It is important to note that these responses may had been triggered by the cellular damage caused by the arthrocentesis performed [17] and the intraarticular injection of a substance/material. The injection of a hemoderived product significantly increase NCC at 6 and 24 h after treatment compared to baseline, which was more significant in the PRP treatments. Similar findings were observed elsewhere [14]. Changes in joint environment were also evidenced by a significant increase in PGE_2_ at T6 which decreased, but did not return to baseline levels. In other reports, PGE_2_ reached peak levels between 3–6 h after intra-articular injection, being only statistically increased up to 48 h after treatment [15, 18]. An important difference is that while aforementioned studies assessed only one joint (metacarpophalangeal) at each time point, this study assessed six joints concurrently in the same horse (middle carpal, tarsocrural and metacarpophalangeal joints). This may have resulted in a global, albeit mild increase in the inflammatory response observed. Additionally, distinct joints may present different responses to inflammation [19].

The differences compared to previous studies [14, 18] in NCC and PGE_2_ levels with the use of PRP, may also be explained by the differences in the methods of preparation and activation for the PRP used. In this study, we used a double centrifugation method, producing a PRP with platelet concentrations 3–4 × above horse’s whole blood (~ 540 × 10^3^ platelets/μL). Both RBC and NCC count in the PRP were below the normal range for the species.^1^ The decrease in NCC, especially neutrophils, observed in the PRP is desirable due to the potential increase in pro-inflammatory cytokines and degradative proteases [20] released by these cells [21].

The platelets in the present study were all activated by freeze- thaw [22, 23], but activation can also occur via injection of resting platelets [14, 18] and/or with the addition of calcium chloride and/or thrombin [14]. Different methods of platelet activation are known to influence the concentration of growth factors present within PRP [24], which may explain the differences in NCC and PGE_2_ observed in equine joints in this study compared to previous reports at the specific time points [14]. Polymorphonuclear (PMN) cells were significantly increased in joints treated with both PRP presentations compared to joints treated with AUTO CS. The significant increase in NCC and PMN for both PRP presentations in comparison to AUTO CS might imply that this product (PRP) is potentially more pro-inflammatory. Although the concentration of leukocytes in PRP was considered low [25] the presence of such cells and platelets (or its fragments), could present a pro-inflammatory effect in joints through the activation of the complement cascade [26, 27]. Additionally, differences in study design and individual variability are factors that could have influenced on the differences observed in this study compared to other (14,18).

Horses in the present study did not show significant change in lameness after CS and PRP injections. The horses did demonstrate other signs of inflammation, such as joint effusion, changes in joint circumference and ROM, although there were no significant changes in heat. Joint effusion significantly increased at T6 hours and although it decreased over time, it did not return to baseline levels. The authors believe that the joint distension observed justifies the decrease in ROM, by affecting proprioceptors within joint capsule [28]. Despite the increased joint effusion in this study and others [14, 18], this response did not result in an increase in lameness.

The fact that the allogeneic products used in this experiment (PRP and CS) did not result in significantly different effects compared to autologous preparations should be noted.

Similar equivalency was also observed in previous experiments in vitro comparing the effects of autologous and allogeneic PRP in fibroblasts [29] and cartilage under inflammatory conditions [11]. Allogeneic PRP was also used to enhance bone healing in a in vivo rabbit model [30]. In this study, we opted for pooling blood-derived products from multiple animals rather than using a single donor. Pooling CS or PRP from different animals leads to a more homogenous product in terms of cytokine composition by reducing individual variability.

Some potential pitfalls of this study should be discussed. To optimize the number of joints used and to reduce the total number of animals, we opted to inject six joints concurrently per horse. This design might have made it difficult to distinguish mild lameness caused by pain in individual joints. However, once we evaluated each individual joint for signs of inflammation (clinical evaluation, cytology and PGE_2_) we were able to distinguish the effects of each treatment. Additionally, we did not perform a cytokine analysis of the CS and PRP used in this study. We would like to emphasize that this was exclusively a safety study and that the evaluation of the composition or the efficacy of the product proposed (ALLO-FD) was not our purpose. Our main focus was to verify if an ALLO-FD version of CS and PRP would trigger any significant inflammatory response compared to the autologous version of the products. Still, detailed investigation of the cytokine profile of the ALLO-FD PRP and CS and further evaluation of the efficacy of this product is warranted in future studies.

## Conclusions

In this study we compared the effects of CS and PRP directly in joints using an equine model and evaluated the effects of a FD-ALLO version of both CS and PRP in joints. Both hemoderived products triggered a self-limiting and mild inflammatory response that was controlled at the termination of the study, not causing significant lameness in horses. The formulation proposed in this project, FD-ALLO demonstrated to be safe to be used in joints, promoting equivalent response compared to the current version of the product used in practice. Further investigation is necessary for evaluation of the clinical efficacy of ALLO-FD PRP and CS and for better understanding the cytokine profile of this product compared to autologous frozen preparations.

## Methods

The main aim of this study was to investigate the effects of a new allogeneic freeze-dried preparation of two biological therapies (PRP and CS) compared to their current version used (fresh/frozen autologous) in equine normal joints. This experiment was a controlled, randomized, blinded in vivo study using the horse as a model. Horses in this study were procured from a vendor and USDA does not regulate the sale of horses at this time if it is not a significant source of income from the vendor to research. After termination of the study and confirmation of soundness, the animals were returned to the vendor.

### Animals

Eight mix-breed healthy horses were used in this experiment. A power analysis was performed using SAS version 9.3, considering alpha 0.05 and actual power of 0.93. The power analysis was based on flexion score data obtained from a previous study evaluating the effects of autologous PRP in equine normal joints [14]. Based on the analysis, four horses were randomly assigned per group (4 for PRP and 4 for CS group) by coin toss. Horses were skeletally mature (2–5 years old) and did not present any clinical or radiographic signs of joint disease. Horses were housed in single outdoor pens throughout the duration of the study with access to a 3-sided shelter and provided fresh water and high-quality grass hay ad libitum. Horses were housed in these conditions with a minimum of 10 days previous to the commencement of the experiment for acclimation. Included horses tested negative for common blood-borne infectious diseases. All methods performed in this experiment were carried out in accordance with the ARRIVE guidelines. The Colorado State University Animal Care and Use Committee (IACUC, 16-6708AA) approved all procedures performed in this experiment. All experiments were performed in accordance with relevant guidelines and regulations of IACUC.

### Conditioned serum preparation

Four horses were randomly assigned to the CS group. After aseptic preparation, a single venipuncture of the jugular vein was performed and 500 mL of blood from each horse was collected. A separate EDTA tube was collected for CBC analysis by automatic cell count.

#### Autologous CS (AUTO CS)

Immediately after collection, blood from each horse was aliquoted into 50 mL conical tubes containing coated beads. The beads were submitted through a process of silanization using a 4% (volume/volume) diethylenetriamine solution in toluene. Blood was incubated for 24 h at 37 ºC. Samples were then centrifuged at 2500 × g for 10 min, and the serum was withdrawn, aliquoted and stored at -80 ºC. For autologous use, aliquots were removed from the freezer and thawed immediately before injection.

#### Allogeneic freeze-dried CS (FD-ALLO CS)

Frozen samples were lyophilized in specific temperature and pressure (< -45 ºC and < 100 mtorr respectively), for 18 h (Virtis Sentry Lyophilizer) and stored at -80ºC until use. Before application, samples from all four horses were diluted in sterile lactated ringer and pooled together to create an allogenic product ready for injection.

### Platelet-rich plasma preparation

Four horses were randomly assigned to the PRP group. Blood (500 mL) was collected from each horse using blood bags containing citrate phosphate dextrose adenine (CPDA) as anti-coagulant. A separate aliquot (EDTA tube) was obtained for hematology as described.

#### Autologous frozen PRP (AUTO PRP)

Blood was immediately aliquoted into 50 mL conical tubes and centrifuged at 200 × g for 18 min at room temperature. The supernatant (platelet-derived product) was collected and centrifuged for 10 min at 1000 × g to create a platelet pellet. The platelet pellets were resuspended in a smaller volume of plasma, concentrating the product threefold. Aliquots were frozen at -80ºC for storage and activation of platelets. For autologous use, the PRP aliquots were thawed and homogenized prior to administration.

#### Allogeneic freeze-dried PRP (FD-ALLO PRP)

Blood was processed as described above, resulting in platelet pellets that were frozen at -80ºC. After freezing, pellets were freeze-dried in a pressure and temperature of at least 100 mtorr and -45 ºC respectively, using a lyophilizer (Virtis Sentry Lyophilizer, Virtis). Samples were freeze-dried for 18 h and stored at -80ºC. Before administration, pellets from the 4 horses were resuspended in sterile lactated ringers and combined to create an allogenic product ready for injection.

### Treatment

**Fig. 4:**
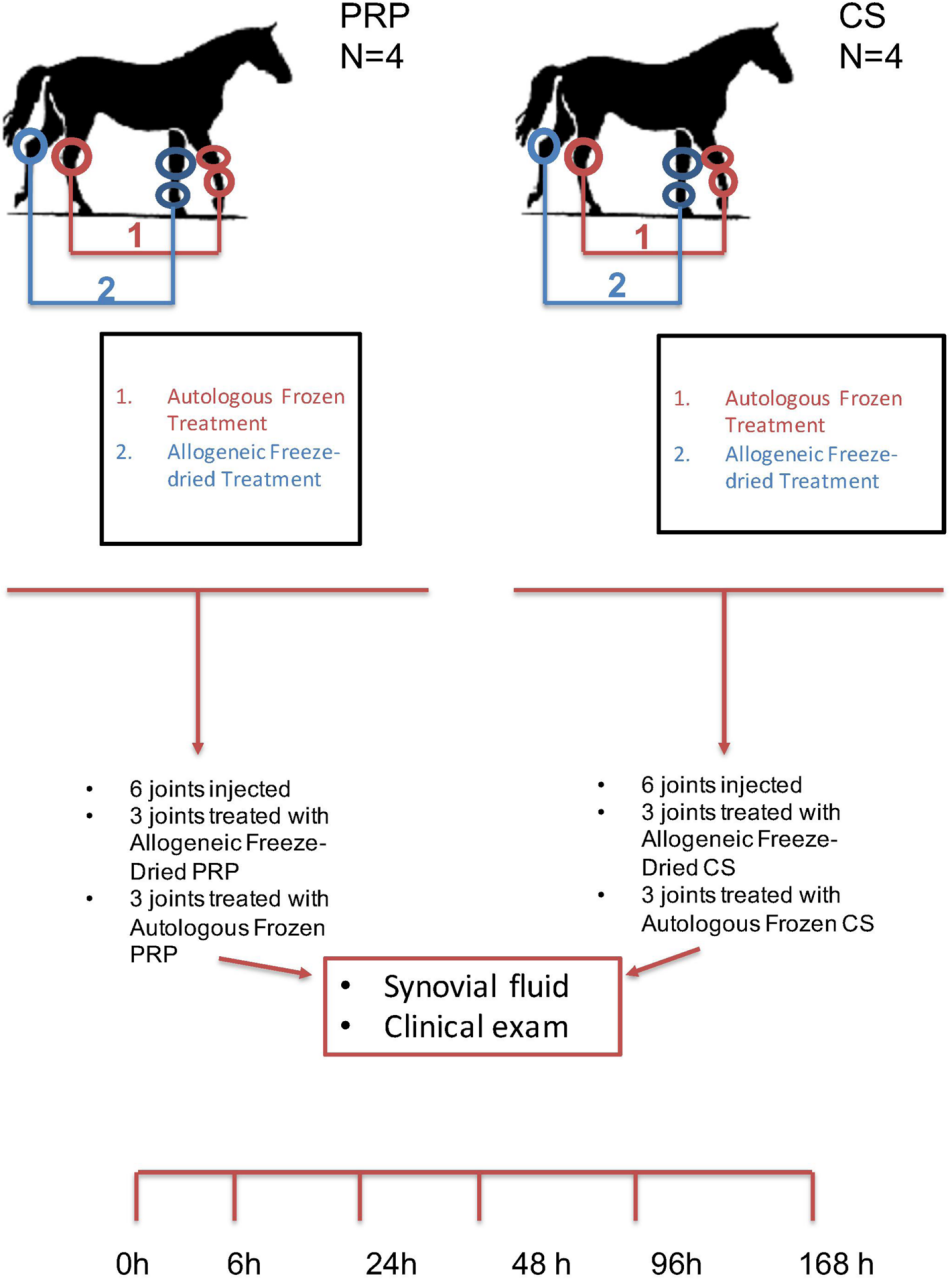
Eight horses were randomly assigned to the platelet-rich plasma (PRP) or conditioned serum (CS) groups (4 horses each group). All horses had the ipsilateral middle carpal, metacarpophalangeal, and tarsocrural joints injected with autologous frozen (AUTO) PRP or CS preparations. The contralateral joints were injected with allogeneic freeze-dried (FD-ALLO) preparation of the same product (i.e., PRP or CS). Horses were clinically evaluated and synovial fluid was collected before treatment (0 h) and at 6, 24, 48, 96 and 168 h after treatment injection

Horses were sedated as needed with 100 mg of xylazine (XylaMed, VetOne), 5 mg of detomidine (Dormosedan, Zoetis) and/or 3 mg butorphanol (Turbogesic, Zoetis) and joints were aseptically prepared. Horses had the ipsilateral middle carpal joint, metacarpophalangeal joint, and tarsocrural joint injected with 6 mL of FD-ALLO product (CS or PRP) while the contralateral joints were injected with 6 mL of AUTO preparation (Fig. 4). A 20G 1.5-inch needle was used for injection. This procedure was performed to optimize the number of joints used in the experiment per horse. Syringes containing the treatments were covered with tape to ensure blindness on application. Because our objective was to evaluate the safety of FD-ALLO compared to hemoderivatives already used clinically, we used AUTO preparations as our control in this experiment. All treatments were injected by the same clinician.

### Clinical examination

Horses were evaluated prior to treatment (T0) as well as 6 (T6), 24 (T24), 48 (T48), 96 (T96), and 168 (T168) hours post-treatment [14]. Vital parameters (temperature, pulse, and respiration) were recorded at each time. The treated joints were evaluated for effusion (none, slight, mild, moderate or severe) and for presence of heat (yes or no). For each treated joint, range of motion (ROM) was measured with a goniometer and circumference was measured using a measuring tape. At each time point, horses were subjectively evaluated for lameness. Each limb was graded on the American Association of Equine Practitioners (AAEP) lameness scale (0: no lameness to 5: non-weight bearing lameness). Response to joint flexion of was graded as well (0-no,1- slight, 2-mild, 3-moderate, 4-severe response to flexion). One blinded examiner performed all of the clinical examinations.

### Synovial fluid collection and analysis

At each time point, synovial fluid was collected from each joint for analysis as described below.

#### Routine synovial fluid analysis

Synovial fluid was subjectively evaluated for color (1-yellow, 2- colorless, 3-straw colored, 4-orange, 5-red), clarity (1-clear, 2-hazy, 3-cloudy) [14], total protein (refractometry) and cytologic evaluation (Advia® 120 hematology analyzer, Siemens Healthineers). Differential counts were performed by a clinical pathologist.

#### PGE_2_ analysis of synovial fluid

PGE_2_ was extracted from synovial fluid as previously described [31]. Briefly, 500 µL of 80% ethanol and 10 µL glacial acetic acid were added to 500 µL of synovial fluid. Samples were incubated for 5 min at room temperature and centrifuged (2,500 × g for 8 min). Content was loaded into C2 minicolumns (Bond Elut C-2 minicolumns, Agilent Technologies) which had been washed multiple times with 10% ethanol under vacuum. After been washed with deionized water and 1 mL of hexane, PGE_2_ was eluded with 1.5 mL of ethyl acetate. Eluded sample was evaporated under a continuous flow of nitrogen, using a multiple-port gas evaporation system (Savant Speed Vac Plus). After evaporation, samples were either stored at -20 °C until assayed or reconstituted in 500 µL assay buffer (PGE_2_ ELISA Kit, Enzo Lifesciences) for analysis by ELISA.

After the extraction of PGE_2_ from the synovial fluids samples_,_ the identification and quantification of PGE_2_ was performed using ELISA, in accordance to the recommendations of manufacturer (PGE_2_ ELISA Kit, Enzo Life Sciences). Briefly, assay buffer was added to the plates into the non-specific binding (NSB) and maximum binding wells (BO) wells. Standards were added to the plate using dilutions from 1000 pg/ml to 7.81 pg/mL, and samples were added in duplicates on the appropriate wells. Conjugate was added to the plate as well as antibody and the plate was incubated at room temperature on a plate shaker for 2 h. After multiple washes, para-nitrophenylphosphate (pNpp) substrate was added to the every well and plate was covered and incubated for 45 min. Stop solution was added and the plate was immediately read at 405 nm, with correction between 570 and 590 nm. The subtraction was performed to correct for optical imperfections in the plate. The optical density of each well was determined using a microplate reader (Spectramax M3, Molecular Devices). Data analysis software (Softmax Pro, Molecular Devices) was used and the standard curve was analysed using a 4 parameter logistic curve.

### Statistical analysis

Results were analyzed by use of a mixed-model ANOVA (PROC GLIMMIX, SAS. version 9.3, SAS Institute Inc.). Student residual plots were used to verify normality log transformation. Hemoderivative (PRP or CS), treatment (AUTO CS, FD-ALLO CS, AUTO PRP or FD-ALLO PRP), time (time of sample collection), allogenicity (AUTO or FD-ALLO), joint (metacarpophalangeal, middle carpal, and tarsocrural joints) and all interactions between main effect variables were considered as fixed effects. Horses were considered a random effect. Results from clinical exam and synovial fluid analysis were considered dependent variables. Restricted Maximum Likelihood was used for estimation and protection against multiple comparisons was achieved using protected F-test. Individual comparisons supported by F-test were performed using least -square means.

For all statistical analyses, a value of P ≤ 0.05 was considered significant. Results are reported as means ± SEM. For the purposes of visual representation, raw data was utilized for presentation of results as mean and SEM, but statistical analysis was based on log transformed data. Differential cell counts were reported as percentages of NCC and statistical analysis was performed using these percentages. Total numbers for each cell type are presented in supplementary tables.

## Supplementary Information

**Additional file 1: Supplementary** **Table 1. **Protein levels in synovial fluid across time prior to (T0) and at T6, T24, T48, T96 and T168 hours after one intra-articular injection of different preparations [AUTO or FD-ALLO] of either PRP or CS. Raw data is presented as mean and SEM. Log-transformed data was used for the statistical analysis with a level of significance set as *P* ≤0.05. Different letters denote statistical significance between treatment groups. **Supplementary** **Table 2. **Nucleated cell count in synovial fluid across time prior to (T0) and at T6, T24, T48, T96 and T168 hours after one intra-articular injection of different preparations [AUTO or FD-ALLO] of two different biologic products (PRP or CS). The table demonstrates the raw data (mean and SEM) used for illustration purposes. Log-transformed data was used for the statistical analysis with a level of significance set as *P* ≤0.05. Different letters denote statistical significance between treatment groups. **Supplementary** **Table 3**. Differential nucleated cell count (NCC) in synovial fluid across time. The table demonstrates the raw data (mean and SEM) of the percentages of NCC and total count for each cell type, used for illustration purposes. Log-transformed data was used when necessary for the statistical analysis with a level of significance set as *P* ≤0.05. Different letters denote statistical significance between different times of the experiment for the same cell type. **Supplementary Table 4**. Differential nucleated cell count (NCC) in synovial fluid by treatment. The table demonstrates the raw data (mean and SEM) of the percentages of NCC and total count for each cell type, used for illustration purposes. Log-transformed data was used when necessary for the statistical analysis with a level of significance set as *P* ≤0.05. Different letters denote statistical significance between different groups for the same cell type. **Supplementary Table 5**. Prostaglandin E_2_ levels in synovial fluid across time prior to (T0) and at T6, T24, T48, T96 and T168 hours after one intra-articular injection of different preparations [AUTO or FD-ALLO] of two different biologic products (PRP or CS). The table demonstrates the raw data (mean and SEM) used for illustration purposes. Different letters denote statistical significance between treatment groups. Log-transformed data was used for the statistical analysis with a level of significance set as *P* ≤0.05.

## Data Availability

The datasets used and/or analyzed during current study are available from the corresponding author on reasonable request.

## Abbreviations

AAEP: American Association of Equine Practicioners
ACS: Autologous conditioned serum
ALLO: Allogeneic
AUTO: Autologous
B0: Maximum binding wells
CBC: Complete blood count
CPDA: Citrate phosphate dextrose adenine
CS: Conditioned serum
EDTA: Ethylenediaminetetraacetic acid
ELISA: Enzyme-linked immunosorbent assay
FD: Freeze-dried
LMN: Large mononuclear cells
NCC: Nucleated cell count
OA: Osteoarthritis
PGE_2_: Prostaglandin E_2_
PMN: Polymorphonuclear cells
pNpp: Para-nitrophenylphosphate
NSB: Non-specific binding
RBC: Red blood cells
ROM: Range of motion
SEM: Standard error of the mean
SMN: Small mononuclear cells

## Footnotes

1 Reference values are based on normalization studies performed at the Colorado State University’s Clinical Pathology Laboratory.
