# Supplementary material for: A safety evaluation of allogeneic freeze-dried platelet-rich plasma or conditioned serum compared to autologous frozen products equivalents in equine healthy joints
Source: BMC Vet Res. 2022 Apr 18;18:141. doi: 10.1186/s12917-022-03225-4 (PMC9014566; doi:10.1186/s12917-022-03225-4)
Supplement: Supplementary file 1 — Additional file 1: Supplementary Table 1. Protein levels in synovial fluid across time prior to (T0) and at T6, T24, T48, T96 and T168 hours after one intra-articular injection of different preparations [AUTO or FD-ALLO] of either PRP or CS. Raw data is presented as mean and SEM. Log-transformed data was used for the statistical analysis with a level of significance set as P ≤0.05. Different letters denote statistical significance between treatment groups. Supplementary Table 2. Nucleated cell count in synovial fluid across time prior to (T0) and at T6, T24, T48, T96 and T168 hours after one intra-articular injection of different preparations [AUTO or FD-ALLO] of two different biologic products (PRP or CS). The table demonstrates the raw data (mean and SEM) used for illustration purposes. Log-transformed data was used for the statistical analysis with a level of significance set as P ≤0.05. Different letters denote statistical significance between treatment groups. Supplementary Table 3. Differential nucleated cell count (NCC) in synovial fluid across time. The table demonstrates the raw data (mean and SEM) of the percentages of NCC and total count for each cell type, used for illustration purposes. Log-transformed data was used when necessary for the statistical analysis with a level of significance set as P ≤0.05. Different letters denote statistical significance between different times of the experiment for the same cell type. Supplementary Table 4. Differential nucleated cell count (NCC) in synovial fluid by treatment. The table demonstrates the raw data (mean and SEM) of the percentages of NCC and total count for each cell type, used for illustration purposes. Log-transformed data was used when necessary for the statistical analysis with a level of significance set as P ≤0.05. Different letters denote statistical significance between different groups for the same cell type. Supplementary Table 5. Prostaglandin E2 levels in synovial fluid across time prior to (T [file 12917_2022_3225_MOESM1_ESM.docx]

**Supplementary** **Table 1:** Protein levels in synovial fluid across time prior to (T0) and at T6, T24, T48, T96 and T168 hours after one intra-articular injection of different preparations [AUTO or FD-ALLO] of either PRP or CS. Raw data is presented as mean and SEM. Log-transformed data was used for the statistical analysis with a level of significance set as P≤0.05. Different letters denote statistical significance between treatment groups.

| Protein (g/dL) | | | | | | |
| --- | --- | --- | --- | --- | --- | --- |
| Time | **0 hour** | **6 hours** | **24 hours** | **48 hours** | **96 hours** | **168 hours** |
| AUTO PRP | 1.2083 ± 0.2763 ^ghij^ | 3.525± 0.2763^a^ | 3.6333± 0.2763^a^ | 2.6083 ± 0.2763^abcd^ | 1.6763 ± 0.281^defgh^ | 1.3583 ± 0.2763^fghi^ |
| FD-ALLO PRP | 1.225 ± 0.2763^ghij^ | 2.7833± 0.2763^abc^ | 3.6833 ± 0.2763^a^ | 2.975 ± 0.2763^ab^ | 1.6917 ± 0.2763^cdefgh^ | 1.375 ± 0.2763^fghi^ |
| AUTO CS | 0.9833 ± 0.2636^ij^ | 2.7833 ± 0.2763^abc^ | 2.3871 ± 0.281^abcde^ | 1.775± 0.2763^bcdefg^ | 1.1 ± 0.2763^hij^ | 1.0833 ± 0.2763^hij^ |
| FD-ALLO CS | 1.1167 ± 0.2763^j^ | 2.9 ± 0.2763^a^ | 2.6417 ± 0.2763^abcd^ | 1.95 ± 0.2763^bcdef^ | 1.6917 ± 0.2763^fghi^ | 1.1667 ± 0.2763^hij^ |

**Supplementary** **Table 2:** Nucleated cell count in synovial fluid across time prior to (T0) and at T6, T24, T48, T96 and T168 hours after one intra-articular injection of different preparations [AUTO or FD-ALLO] of two different biologic products (PRP or CS). The table demonstrates the raw data (mean and SEM) used for illustration purposes. Log-transformed data was used for the statistical analysis with a level of significance set as P≤0.05. Different letters denote statistical significance between treatment groups.

| Nucleated cell count (cells/µL) | | | |  |  |  |  |  |
| --- | --- | --- | --- | --- | --- | --- | --- | --- |
| Treatment/Time | **0 hour** | | **6 hours** | | **24 hours** | **48 hours** | **96 hours** | **168 hours** |
| AUTO PRP | 150± 2719.34^l^ | 40825± 2719.34^ba^ | | | 22475 ± 2719.34 ^abc^ | 7508.33 ±2719.34 ^de^ | 2020.64 ± 2839.17^gh^ | 533.33 ± 2719.34^ijk^ |
| FD-ALLO PRP | 133.33 ± 2719.34^l^ | 33033± 2719.34^a^ | | | 38892 ± 2719.34^ab^ | 9966.67 ± 2719.34^de^ | 1533.33± 2719.34^gh^ | 716.67± 2719.34^ij^ |
| AUTO CS | 250 ± 2719.34^l^ | 6250±  2719.34 ^g^ | | | 7491.46± 2839.17^ef^ | 2916.67 ± 2719.34^g^ | 1141.67 ±2719.34 ^ih^ | 533.33 ± 2719.34^jk^ |
| F-FD ALLO CS | 275± 2719.34^lk^ | 11300± 2719.34 ^bcd^ | | | 10200± 2719.34 ^cd^ | 3550 ± 2719.34 ^gf^ | 991.67 ± 2719.34^ih^ | 600 ± 2719.34^ij^ |

**Supplementary** **Table 3**: Differential nucleated cell count (NCC) in synovial fluid across time. The table demonstrates the raw data (mean and SEM) of the percentages of NCC and total count for each cell type, used for illustration purposes. Log-transformed data was used when necessary for the statistical analysis with a level of significance set as P≤0.05. Different letters denote statistical significance between different times of the experiment for the same cell type.

| Large mononuclear cells | 0 hour | 6 hours | 24 hours | 48 hours | 96 hours | 168 hours |
| --- | --- | --- | --- | --- | --- | --- |
| % of NCC | 48.85^b^ | 13.56^d^ | 34.8295^c^ | 51.0625^b^ | 66.167^a^ | 66.3333^a^ |
| SEM (% of NCC) | 3.2712 | 3.2712 | 3.3018 | 3.2712 | 3.3018 | 3.2712 |
| Total number (cells/µL) | 89.08 | 2041.54 | 4756.96 | 2644.60 | 1044.73 | 406.42 |
| SEM (cells/µL) | 15.02 | 315.52 | 582.03 | 256.86 | 110.71 | 52.29 |
| Small mononuclear cells |  |  |  |  |  |  |
| % of NCC | 42.5833^f^ | 5.5625^e^ | 2.4154^d^ | 8.875^c^ | 11.6072^bc^ | 20.3542^ab^ |
| SEM (% of NCC) | 5.5501 | 5.5501 | 5.6197 | 5.5501 | 5.6197 | 5.5501 |
| Total number (cells/ µL) | 75.10 | 953.85 | 515.75 | 364.83 | 161.33 | 116.46 |
| SEM (cells/ µL) | 9.43 | 330.87 | 95.62 | 46.18 | 16.26 | 15.81 |
| Polymorphonuclear cells |  |  | | | |  |
| % of NCC | 7.2917^d^ | 80.6667^a^ | 57.7869^b^ | 40^c^ | 9.4113^d^ | 12.5^d^ |
| SEM (% of NCC) | 2.3974 | 2.3974 | 2.4234 | 2.3974 | 2.4234 | 2.3974 |
| Total number (cells/ µL) | 17.06 | 19856.69 | 14227.65 | 2974.35 | 116.85 | 72.96 |
| SEM (cells/ µL) | 6.04 | 2779.33 | 3013.05 | 496.98 | 20.28 | 14.94 |

**Supplementary Table 4**: Differential nucleated cell count (NCC) in synovial fluid by treatment. The table demonstrates the raw data (mean and SEM) of the percentages of NCC and total count for each cell type, used for illustration purposes. Log-transformed data was used when necessary for the statistical analysis with a level of significance set as P≤0.05. Different letters denote statistical significance between different groups for the same cell type.

|  | AUTO PRP | | FD-ALLO PRP | AUTO CS | FD-ALLO CS |
| --- | --- | --- | --- | --- | --- |
| Large mononuclear  cells | | | | | |
| % of NCC | 40.047^b^ | 35.7372^b^ | | 55.0657^a^ | 50.8655^a^ |
| SEM (% of NCC) | 3.4542 | 3.4459 | | 3.4542 | 3.4459 |
| Total number (cells/µL) | 2308.33 | 2183.80 | | 1338.94 | 1491.14 |
| SEM (cells/ µL) | 362.69 | 352.49 | | 250.19 | 203.1 |
| Small mononuclear  cells | | | | | |
| % of NCC | 20.6598 ^a^ | 42.45^a^ | | 21.17^a^ | 17.52^a^ |
| SEM (% of NCC) | 4.96 | 4.94 | | 4.96 | 17.52 |
| Total number (cells/ µL) | 605.03 | 490.94 | | 165.79 | 196.46 |
| SEM (cells/uL) | 203.35 | 119.18 | | 25.23 | 24.45 |
| Polymorphonuclear  cells | | | | | |
| % of NCC | 35.4037^a^ | 35.6329^a^ | | 21.3175^b^ | 29.4226^a^ |
| SEM (% of NCC) | 2.3652 | 2.3575 | | 2.3652 | 2.3575 |
| Total number (cells/ µL) | 9271.67 | 11371.08 | | 1402.44 | 2798.51 |
| SEM (cells/ µL) | 2010.56 | 2372.64 | | 355.157 | 493.08 |

**Supplementary Table 5**: Prostaglandin E_2_ levels in synovial fluid across time prior to (T0) and at T6, T24, T48, T96 and T168 hours after one intra-articular injection of different preparations [AUTO or FD-ALLO] of two different biologic products (PRP or CS). The table demonstrates the raw data (mean and SEM) used for illustration purposes. Different letters denote statistical significance between treatment groups. Log-transformed data was used for the statistical analysis with a level of significance set as P≤0.05.

| PGE_2_ (pg/mL) | | | | | | |
| --- | --- | --- | --- | --- | --- | --- |
| Time | **0 hour** | **6 hours** | **24 hours** | **48 hours** | **96 hours** | **168 hours** |
| AUTO PRP | 63.69 ±  31.46^cde^ | 105.27 ± 32.33^abcd^ | 85.71 ± 29.68^bcde^ | 92.11 ± 33.22^abcd^ | 31.7 ±31.46^de^ | 44.52 ± 29.68^e^ |
| FD-ALLO PRP | 100.12 ± 34.79^abcd^ | 101.06 ± 30.59^abcd^ | 78.56 ± 29.68^bcde^ | 66.35 ± 30.59^bcde^ | 110.78 ± 33.22^abcde^ | 83.8 ± 29.68^bcde^ |
| AUTO CS | 111.36 ± 29.68^abcd^ | 153.26 ± 29.68^ab^ | 135.73 ±  30.59^abc^ | 138.04 ± 30.59^abcd^ | 172.66 ± 30.59^ab^ | 132 ± 31.52^abc^ |
| FD-ALLO CS | 89.28 ±29.68^abcd^ | 130.57 ± 29.68^abc^ | 169.6 ±  29.68^a^ | 166.06 ± 29.68^abc^ | 166.09 ± 30.59^abcd^ | 88.012 ± 30.59^abcde^ |
